# Immunotherapy and brain metastasis in lung cancer: connecting bench side science to the clinic

**DOI:** 10.3389/fimmu.2023.1221097

**Published:** 2023-10-09

**Authors:** Alejandro Rios-Hoyo, Edurne Arriola

**Affiliations:** ^1^ Yale Cancer Center, Yale School of Medicine, Yale University, New Haven, CT, United States; ^2^ Department of Medical Oncology, Hospital del Mar-CIBERONC (Centro de Investigación Biomédica en Red de Oncología), Barcelona, Spain; ^3^ Cancer Research Program, Institut Hospital del Mar d’Investigacions Mèdiques (IMIM), Barcelona, Spain

**Keywords:** brain metastases, NSCLC, SCLC, immunotherapy, immune microenvironment

## Abstract

Brain metastases (BMs) are the most common form of intracranial malignant neoplasms in adults, with a profound impact on quality of life and traditionally associated with a dismal prognosis. Lung cancer accounts for approximately 40%–50% of BM across different tumors. The process leading to BMs is complex and includes local invasion, intravasation, tumor cells circulation into the bloodstream, disruption of the blood–brain barrier, extravasation of tumor cells into the brain parenchyma, and interaction with cells of the brain microenvironment, among others. Once the tumor cells have seeded in the brain parenchyma, they encounter different glial cells of the brain, as well as immune cells. The interaction between these cells and tumor cells is complex and is associated with both antitumoral and protumoral effects. To overcome the lethal prognosis associated with BMs, different treatment strategies have been developed, such as immunotherapy with immune checkpoint inhibitors, particularly inhibitors of the PD-1/PD-L1 axis, which have demonstrated to be an effective treatment in both non-small cell lung cancer and small cell lung cancer. These antibodies have shown to be effective in the treatment of BM, alone or in combination with chemotherapy or radiotherapy. However, many unsolved questions remain to be answered, such as the sequencing of immunotherapy and radiotherapy, the optimal management in symptomatic BMs, the role of the addition of anti–CTLA-4 antibodies, and so forth. The complexity in the management of BMs in the era of immunotherapy requires a multidisciplinary approach to adequately treat this devastating event. The aim of this review is to summarize evidence regarding epidemiology of BM, its pathophysiology, current approach to treatment strategies, as well as future perspectives.

## Background and epidemiology

Brain metastases (BMs) are the most common form of intracranial malignant neoplasms in adults and are associated with a dismal prognosis (1–3). Lung cancer is the most frequent primary tumor to develop BM (4) and globally accounts for approximately 40%–50% of all BMs (1, 5). Different risk factors have been associated to lung cancer, namely, cigarette smoking history, radon exposure, and occupational exposure to agents such as arsenic, asbestos, beryllium, cadmium, chromium, coal smoke, diesel fumes, nickel, silica, soot, and uranium. Other risk factors include history of prior malignancies such as lymphoma or head and neck cancers, history of chronic obstructive pulmonary disease, or pulmonary fibrosis (6). Among the different subtypes of lung cancer, it is estimated that up to 22%–57% of patients with non-small cell lung cancer (NSCLC) will develop BMs at some point of the history of their disease (7–10). Overall, 20% of these patients present BM at diagnosis and up to 57% develop BM over the period of the disease (7, 11). The prevalence of BMs in a subset of patients with NSCLC and oncogenic drivers can be higher in some cases and increase throughout the course of their disease. These patients experience different rates of BM at baseline and through the history of their disease, as follows for the most frequent oncogenic drivers: *EGFR* mutations are 24.4% and 52.9% (12), *ALK* rearrangements are 23.8% and 72% (12, 13), and *ROS1* rearrangements are 19.4% and 34% (13). Regarding the less common genomic alterations, the percentages for baseline BM and throughout the history of the disease are as follows: *BRAF* V600E mutations are 31% (14) and not reported (possibly due to the low prevalence of *BRAF* mutations in NSCLC), *HER2* mutations are 14% and 28% (15, 16), *RET* rearrangements are 25% and 46% (17), and *KRAS* mutations are (G12C and non-G12C) 24% and 35.1% (15, 18). Some patients with oncogenic drivers and BMs benefit from targeted therapies with tyrosine kinase inhibitors, which can surpass the blood–brain barrier (BBB), as evidenced by significant intracranial response rates in different clinical trials (19–22). For patients with small cell lung cancer (SCLC), it is estimated that 10%–20% of patients will present with BM at diagnosis, and up to 50%–80% of patients will develop BMs at some point of the history of their disease (23–26).

Detecting BM at baseline has been associated to a worse prognosis, and developing BM during ongoing therapies reflects tumor progression and resistance to treatment, associated to the disruption of the physiologic BBB.

Current guidelines recommend the use of brain magnetic resonance imaging (MRI) with contrast or, when not possible, brain computed tomography with contrast for patients with NSCLC and SCLC stages II–IV to detect BM (27, 28). Although BMs have a negative impact in survival, in recent years, patients’ outcomes have improved significantly (29, 30). Some of the treatments that have contributed to improving survival of these patients include surgery, stereotactic radiosurgery, whole-brain radiation therapy, prophylactic cranial irradiation, and the addition of better systemic therapies (10, 31–33). To develop effective treatment strategies, it is necessary to understand the genomic and molecular mechanisms leading to BM. The purpose of this review is to provide an integrated synthesis of the epidemiology of BM in lung cancer patients. To describe its pathophysiology and molecular mechanisms, as well as its genomic landscape. Furthermore, we intend to provide a cohesive summary of the clinical evidence for the different treatment strategies, focusing on immunotherapy, and immunotherapy based combinations. Finally, we present future perspectives for the management of BMs.

## The blood–brain barrier, the blood–tumor barrier, and the premetastatic niche

**Figure 1:**
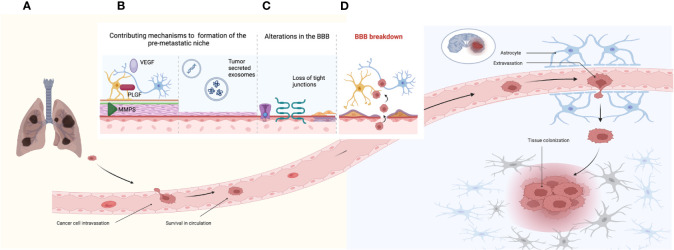
Establishment of brain metastases. **(A)** Mechanisms of tumor cell invasion, intravasation, survival in circulation, extravasation, and tissue colonization. **(B)** Mechanisms associated to the pre-metastatic niche including factors such as VEGF, PLGF, and MMP (matrix metalloproteinases). **(B)** Tumor-secreted exosomes containing nucleic acids such as miRNAs and lncRNAs, as well as proteins and lipids. **(C)** Contributing factors that increase permeability of the BBB including alterations in channel transporters, loss of tight junctions, and collagen produced by pericytes. **(D)** The BBB breakdown and the establishment of the BTB reflected by tumor cell extravasation into the brain parenchyma.

The mechanisms leading to BMs are complex and different mechanisms are required; among them, local invasion, intravasation, survival in circulation, extravasation, and tissue colonization represent the most remarkable (**Figure 1A**) (34, 35). The most difficult steps in this scenario are extravasation and colonization, since the BBB must be bypassed (35). Under physiological conditions, the BBB regulates the homeostasis of the central nervous system (CNS); it provides the brain with nutrients, limits the transportation of ionic substances and other molecules, and functions as a critical obstacle for drug transportation to the brain (36–38). Key components of the BBB include endothelial cells, pericytes, astrocytes, and the extracellular matrix. Endothelial cells form tight junctions that control transportation and entry of different molecules, allowing a restricted permeability of the BBB (37, 39, 40). These cells secrete growth molecules such as PDGF-β, TGF-β, and Vascular Endothelial Growth Factor (VEGF), which modulate pericytes (40). Pericytes wrap blood vessels through their end feet processes and regulate the vascular function. They are involved in angiogenesis, neovascularization, and antigen cell presentation (under certain conditions), as well as communicating with astrocytes within the neurovascular unit (41–43). Astrocytes are necessary for different metabolic processes in the brain, including metabolization of different substances and production of antioxidants, as well as regulation of signaling pathways. They contribute to the function of the BBB and secrete growth factors targeted to the endothelial cells (40). Another important component of the BBB is the extracellular matrix, composed of the basal lamina that surrounds the BBB (43). Disruption of the extracellular matrix has been associated with an increased permeability of the BBB and as a consequence development of BM (40). In addition to the BBB, other components of the CNS environment include oligodendrocytes and microglia; the latter are the innate macrophage cells of the brain and can exert disrupting effects in the BBB, facilitating the formation of BM (43, 44).

Prior to the development of BM, an adequate microenvironment must be secured for the establishment of the metastatic cells; this pretumoral microenvironment is referred to as the premetastatic niche (**Figures 1A, B**) (45). Different molecules contribute to the formation of the premetastatic niche, as well as to the disruption of the BBB, including VEGF, PLGF, and matrix metalloproteinases, which elicit changes in the tight junctions leading to a disruption of BBB (46–48). Other contributing mechanisms accounting for the conditioning of the premetastatic niche are tumor-secreted exosomes, which are vesicles surrounded by a bilayer of lipids. Exosomes contain nucleic acids, lipids, and proteins, which can regulate integrins and modulate glucose uptake in astrocytes (49, 50). MicroRNAs (miRNAs) are a type of nucleic acids contained in exosomes; these are characterized by being small non-coding RNAs that can alter gene expression. Different miRNAs have been associated to the development of BM, such as miR-378, which is relevant to the promotion of cell migration, invasion, and angiogenesis via upregulation of *PRKCA* (51–53). A different type of nucleic acids involved in BMs are long non-coding RNAs (lncRNAs), which are RNA sequences longer than 200 nucleotides, which are not translated into functioning proteins (54). The lncRNA MALTA1 has been associated with promoting BMs by inducing epithelial to mesenchymal transition (EMT), whereas the lncRNA HOTAIR contributed to BMs by increasing cell migration and anchorage (55, 56). In addition, contributing factors to the disruption of the BBB can be attributed to endothelial cells, which express different adhesion molecules prompted by tumor cells, facilitating invasion conditions for these tumor cells (57).

Once the BBB has been disrupted, the blood–tumor barrier (BTB) is established (**Figures 1C, D**). The BTB is characterized by alterations in the transportation of molecules, changes in protein expression profiles involving transportation channels, and a heterogeneous permeability to different molecules (44). However, this increased permeability does not necessarily translate into a more favorable anti-cancer drug delivery (44). As a former BBB, the BTB presents similar components, such as endothelial cells, which in the BTB scenario upregulate TNF receptors 1 and 2 and develop alterations in cell adhesion proteins leading to a loss of tight junctions. The loss of endothelial tight junctions increases paracellular permeability and downregulates specific transporters related to influx and efflux of molecules (58). Other cells that contribute to the disruption of the BBB are pericytes, which locate around blood vessels, forming double layers of cells and producing collagen, which leads to a thickening of blood vessels (59). An upregulation in the expression of VEGF in the BTB has been described; however, it does not always translate into an effective formation of blood vessels (60, 61). Other components associated with the establishment of the BTB include the production of inflammatory molecules, membrane proteins, and growth factors, such as claudin-5 (62, 63). Following the disruption of the BBB and the formation of the BTB, metastatic cells can then settle in the brain. Once they are established, tumor cells interact with the innate microenvironment of the brain. The tumor microenvironment is a complex ecosystem composed of tumor cells, as well as non-malignant cell types such as endothelial cells, astrocytes, pericytes, and immune cells (64).

## The tumor microenvironment of brain metastases

Once, in the brain parenchyma, the tumor cells trigger an inflammatory response led by astrocytes, microglia, and other immune cells (65). Astrocytes are able to exert both pro-tumoral and antitumoral functions (66). Initially astrocytes exert antitumoral effects through the production of nitric oxide and plasminogen activator, as well as creating an astrocytic wall, which separates the metastasis from the brain parenchyma (67–69). Plasminogen activator triggers the production of plasmin, which activates the FasL pathway and deactivates L1CAM, ultimately suppressing BM. This results in a death signal for cancer cells and inhibition of metastatic growth. In response to the deleterious effects exerted by plasmin to the tumor cells, the latter secrete serpins which elicit inhibitory effects against the plasminogen activator and therefore counteract the antitumoral functions of astrocytes (68, 70). A worth noting pro-tumoral effect of astrocytes is the ability to establish gap junctions with tumor cells, facilitating the transfer of cGAMP to astrocytes. Consequently, the STING pathway is activated, leading to the production of cytokines such as IFN-α and TNF, promoting tumor cell growth and chemoresistance. Additionally, these gap junctions hinder the uptake of calcium by tumors, further contributing to tumor growth and chemoresistance (71–73). In addition, reactive astrocytes (RAs) are astrocytes that undergo changes in their molecular, morphological, and functional states as response to pathologic situations in the surrounding environment (74). Tumor cells promote the activation of STAT3 in RA, contributing to the modulation of the immune system, and promote the establishment of a pro-metastatic environment through a decrease in the activation of CD8+ T cells (75). RA may also contribute to tumor proliferation through the secretion of IL-6, TNF- α, and IL-1β (69).

**Figure 2:**
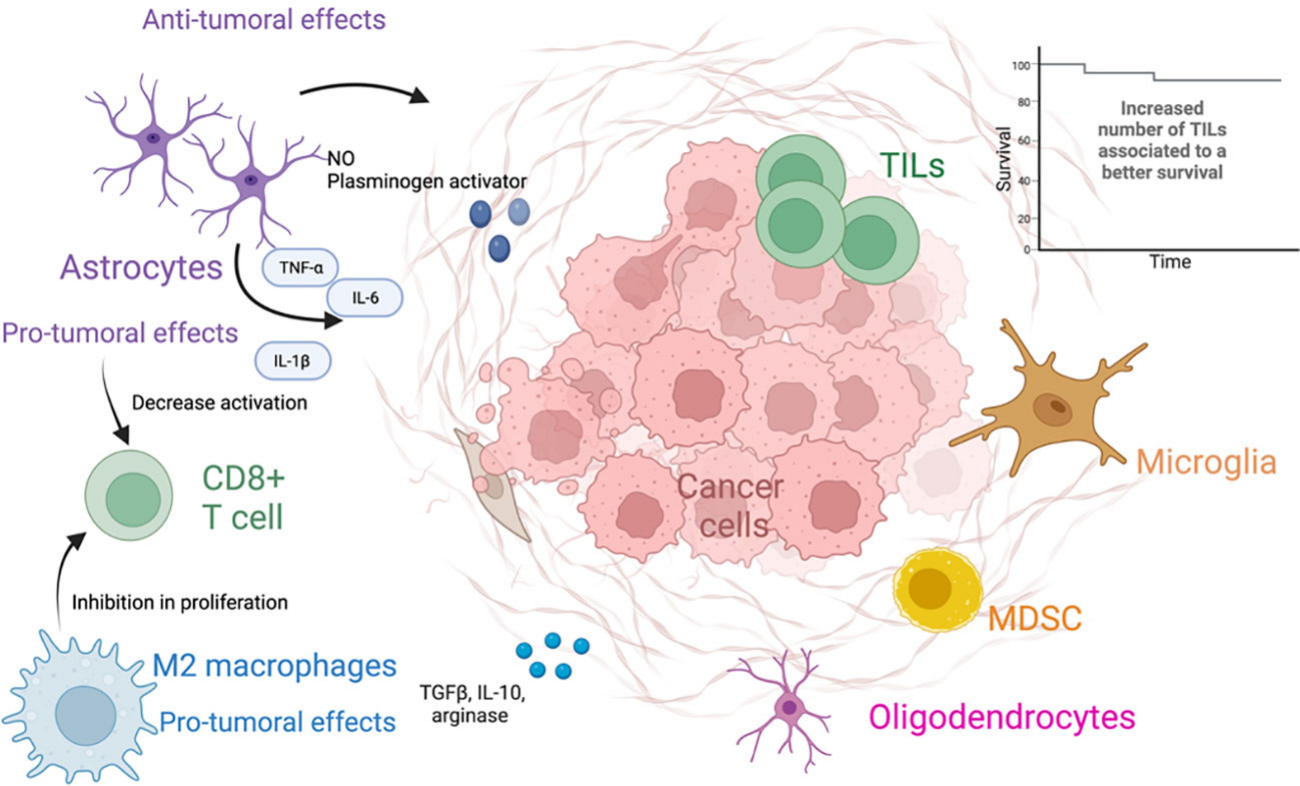
The tumor microenvironment of brain metastases. The figure different types of cells present in the tumor microenvironment and its anti- and protumoral effects.

The microglia are the resident macrophages of the CNS and not derived from the bone marrow. However, bone marrow–derived macrophages can reach the CNS when the BBB is disrupted, as a response to disturbances in the CNS. Both of them share lineage and activation markers, and in the context of malignant neoplasms, these cells are referred to as tumor-associated macrophages (TAMs) (76, 77). These macrophages can be subsequently divided according to their functional characteristics; M1-like macrophages are usually described to be proinflammatory and are stimulated by Toll-like receptor ligands, as well as IFN-γ and TNF-α. They are thought to exert tumor-suppressive functions, through the production of IL-1, IL-12, and nitric oxide, among other factors (78, 79). On the other hand, M2-like macrophages are usually considered anti-inflammatory; are activated by IL-4 and IL-13; and produce molecules such as TGF-β, arginase, IL-10, and profibrotic factors. M2-like macrophages are associated with tumor promoting functions through the inhibition of CD8+ T-cell proliferation (78, 79) (**Figure 2**).

Tumor-infiltrating lymphocytes (TILs) in BMs are usually composed of different subsets of CD8+ T cells and CD4+ T cells, including T-regulatory cells (Tregs) (80). TILs in BM have been associated to peritumoral edema in the brain. Some of the main TILs present in BMs include CD3+, CD8+ cytotoxic TILs, and CD45RO+. A high density of these subpopulations has been associated with a prolonged overall survival (OS) (81). For patients with metastatic SCLC, resection of BM is not a standard of practice; thus, characterization of BM in these patients is not often achieved (82). However, a study in 32 patients with SCLC and resected BM studied the immune microenvironment of these metastases. CD3+ TILs, CD8+ TILs, CD45RO+ TILs, and FOXP3 + TILs were present in over 45% of the studied specimens. PD-L1 expression was positive in 75% of the studied BM, whereas for TILs and TAMs, the percentage of expression was 25% and 28.1%, correspondingly. The expression of PD-L1 on TILs was associated with improved survival (83). In a different study including 49 patients with NSCLC evaluated peripheral blood immune cells, including immunosuppressive monocytes, myeloid-derived suppressor cells, and Tregs. The study found that patients with BM had an increased number of PD-L1+ monocytes, myeloid-derived suppressor cells, and Tregs in peripheral blood, compared to patients who did not have BM and healthy controls. The study also found that patients with elevated numbers of peripheral myeloid PD-L1–expressing cells were associated with a worse progression-free survival and OS (84).

## Comparing the tumor microenvironment and genomic landscape of brain metastases with matched primary tumors

Different studies have evaluated primary NSCLC tumors and paired BM to assess the tumor microenvironment, as well as the expression and mutational profiles in both samples. A study in 39 patients with NSCLC and resected BM performed DNA sequencing of 158 hotspot mutations, TCR-β sequencing analysis, as well as immunohistochemistry staining to assess differences and similarities between the studied specimens. The variant allelic frequency of mutated genes was higher in BM compared to primary tumors, with a median difference of 21.6%. BM showed an inhibition of dendritic cell maturation, Th1 immune response, as well as leukocyte extravasation signaling, and a reduced expression of the adhesion molecule VCAM1. Furthermore, a bioinformatic evaluation of the immune subpopulations based on gene expression detected that the abundance of Th1 or CD8 T genes was lower in BM compared to primary tumors, which was associated with reduced levels of CD8+ T, dendritic cells, and macrophages in BM. The T-cell richness and T-cell densities were significantly lower in BM; however, levels of bone marrow–derived M2-like macrophages were higher in BM. The results from this study suggest that the immune tumor microenvironment of BM presents increased immunosuppressive features compared to the one in primary tumors (85). Another study in 23 patients with matched primary NSCLC and BM identified that PD-L1 expression in tumor cells was higher and CD8+ TILS was lower in BM compared to primary tumors. However, patients categorized as CD8 high stromal TILs in BM had a trend toward a better OS, compared to those with CD8 low stromal TILs (86). In addition, a study involving 43 NSCLC patients evaluated samples of matched primary tumor and BM, identifying higher numbers of neutrophils, CD4+ T cells and dendritic cells, and lower fractions of M1-like macrophages and Tregs in BM. The study also detected a downregulation in the expression of CTLA-4 in BM, as well as lower expression of PD-L1 and CD8A between primary tumors and BM. Interestingly, an analysis between patients with metachronic tumors who received adjuvant treatment and later had a brain relapse, compared to those who were not exposed to prior treatments and later had a brain relapse, showed no differences except for the cell fraction of natural killer cells, which were higher in the group that received adjuvant treatment. Furthermore, this study identified that the immune microenvironment differed between primary tumors and BM in the metachronic group compared to the synchronic group. The results of this study concur with the previous ones in detecting an immunosuppressed tumor microenvironment in BM, compared to the primary tumor (87). A study in 34 patients with NSCLC and BM, who underwent surgical resection or autopsy, compared primary tumors with BM. This study found that only CD204+ cells, corresponding with macrophages, were higher in tumor areas of BM compared to the primary tumor, whereas CD4+ T cells, CD8+ T cells, and CD+ FOXP3 T cells were higher in the primary tumor. The study found that higher densities of CD4+ T cells and CD8+ T cells in both BM and the stromal areas of BM were associated with a higher OS (88).

A study compared the expression of PD-L1 in primary and metastatic lesions; 747 BM samples from patients with NSCLC were included. The study compared the expression of PD-L1 between primary tumors and BM and found a discordance in the expression of PD-L1. BM had a low or negative expression of the biomarker, highlighting the heterogeneity of PD-L1 according to the tumor sample site (89).

Other studies have focused on genomic profiling of NSCLC primary tumors and BM. As such, a study found that non-synonymous tumor mutational burden (TMB) was higher in BM compared to primary tumors; however, the neoantigen load was similar in BM and primary tumors. The study also found a reduced T-cell heterogeneity in BM associated to a immunosuppressive tumor microenvironment (90). A different study profiled BM and primary tumor samples from patients with NSCLC and identified a discrepancy in the mutational landscape between the samples, with a median of 8.3% of shared genetic mutations and higher numbers of somatic mutations in BM. Different pathways were enriched in BM compared to primary tumors, including invasion/metastasis and metabolic associated pathways. The study also found a phylogenetic divergence between BM and primary tumors, indicating parallel progression models for each of the lesions (91). Another study in 11 patients with metastatic lung adenocarcinoma, assessed the mutational landscape of primary tumors and BM. The study found that more unique mutations were present in BM, and identified mutations in *FAM129C* and *ADAMTS* present specifically in BM. Alterations in the APOBEC signature were also higher in BM compared to primary tumors (92). A different study performed in samples from 2,309 patients with lung adenocarcinoma, including 238 samples of CNS metastases identified genes associated to the development of BMs. Alterations in the Hippo pathway were associated to a shorter time to developing CNS metastases, and *STK11* alterations were more prevalent in CNS metastases (93). Another study evaluated 73 samples of BM from lung adenocarcinoma and compared them against a control population of 503 primary lung adenocarcinoma tumors. The study found different genes that were considered as candidates for the development of BM through the identification of regions with higher amplification frequencies or deletions. These genes included *MYC*, *YAP1*, *MMP13*, and *CDKN2A/B* (94). A study in 51 patients with lung adenocarcinoma, as well as squamous cell carcinoma, identified that BM had a higher number of somatic copy number alterations. Gene alterations associated with BM driving mechanisms include *CDK12*, *DDR2*, *ERBB2*, and *NTRK1* (95). A different study in 54 paired samples from BM and NSCLC patients identified that most driver alterations were present both in primary tumor and BM. Private alterations to one or the other sites were present in 22%–26% of the cases. *KRAS* mutations were more frequent in primary tumors that developed BM compared to data from The Cancer Genome Atlas (CTGA) (96). The preceding studies highlight that BM are composed of an immunosuppressive tumor environment, which has been associated to a deleterious prognosis. Furthermore, different mutational patterns in primary tumors versus BMs represent the tumor heterogeneity between the two tumor sites and could in part explain the development of BM and the different response to therapy. The most relevant results from these studies are presented in **Table 1**.

**Table 1:** Comparison of differentially expressed and regulated genes, molecules, signatures, and cell in primary tumor versus brain metastases.

Increased abundance or expression in primary tumor	Increased abundance or expression in BM	Decreased abundance or expression in BM
CD4+ T cells	Mutations in *STK11*, *FAM129C, ADAMTS*, and *APOBEC* signature	Th1 and CD8 T genes
CD8+ T cells	TMB	Expression of VCAM1
CD+ FOXP3	Higher number of somatic mutations	Leukocyte extravasation signaling
PD-L1 expression*	Higher variant allelic frequency of mutated genes.	Th1 immune response
	Invasion, metastasis, and metabolic-associated pathways	Reduced T cell heterogeneity
	Alterations in Hippo pathway	Dendritic cell maturation
	PD-L1 expression*	CD8+ T cells
		Macrophages (not TAMs)
		CTLA-4

*Discordant results.

## Approach to treatment strategies

Different strategies have been used to approach treatment of BM; however, there is not a definitive consensus regarding the sequence of treatment. Neurosurgery should be considered in patients with large tumors that exert a mass effect as well as in patients with minimal intracranial disease. Patients with multiple BM or a single BM could be considered for whole brain radiotherapy (WBRT) or stereotactic radio surgery (SRS) or stereotactic radiotherapy (SRT). However, radiotherapy is not recommended for patients with a Karnofsky Performance Status (KPS) ≤50 or <70 and no systematic therapy options. The use of WBRT, SRS, or SRT can also be considered for patients with a KPS <70 or with comorbidities impeding neurosurgery. Combination of SRS plus WBRT (with our radioprotective strategies) is associated with higher rates of cognitive impairment. Thus, the combination of SRS with WBRT could be used with hippocampal avoidance or administration of memantine to decrease neurological toxicity. In some instances, local treatment can be deferred to avoid potential adverse effects. Situations that might prompt to a deferral for local treatments include asymptomatic BM and a “favorable” location of the BM and systemic treatments with high CNS penetrance and responses (i.e., third-generation EGFR tyrosine kinase inhibitors) (97). Following local treatment strategies, a systemic treatment should be considered, such as immunotherapy, tyrosine kinase inhibitors, chemotherapy, or combination therapies. It is worth noting that administering concurrent radiotherapy to the brain and chemotherapy is not recommended, since it can trigger the development of cognitive impairment, white matter damage, and radionecrosis (98–100).

More recently, in the era of immunotherapy, PD-1 or PD-L1 inhibitors alone or in combination with chemotherapy or anti–CTLA-4 agents have demonstrated activity both NSCLC and SCLC patients, with BM (27, 82, 101–103). The use of Immune checkpoint-inihbitors (ICI) is an attractive strategy to stimulate the immune response of the immunosuppressed tumor microenvironment in BM (104, 105). ICI has demonstrated to be effective in the treatment of BM in other tumors including melanoma, which is coined one of the pioneers of immunotherapy. Clinical trials have demonstrated that patients with asymptomatic BM benefit from the nivolumab–ipilimumab combination, reporting an intracranial benefit of 57%, and an OS at 3 years of 71.9% in this population (106–108). Different clinical trials and retrospective studies have evaluated the effect of ICI alone or in combination with chemotherapy or radiotherapy in BM from lung cancer; these studies are summarized in **Table 2**. The current use of immunotherapies is based on regulation from medical agencies, as well as clinical guidelines. Current approvals can be summarized as follows: for tumors with a PD-L1 expression 0%–49% treatment recommendations include platinum-based chemotherapy with anti–PD-1/PD-L1 ± ipilimumab ± bevacizumab (141). Tumors with a PD-L1 expression ≥50% benefit from the prior mentioned regimens or anti–PD-1/PD-L1 agents in monotherapy. For patients with SCLC, the current recommendations are the use of chemoimmunotherapy in the first line of treatment (82).

**Table 2:** Clinical evidence of the use of ICI in brain metastases from lung cancer.

Study	BM population included	Prospec-tive (P) or Retrospec-tive (R)	Intervention	Treat-ment Line	Control arm	Num. of patients with BM	ORR (Num. %)	ICR (Num. %)	PFS for patients with BM	OS for patients with BM	Ref
Studies with ICI monotherapy in NSCLC											
OAK study	NSCLC pts with treated asymptomatic CNS BM	P	Atezolizumab (At)	> 1	Docetaxel (Do)	61 At, and 62 Do	13.7 for At, and 11.8 for Do	NR	NR	16 m for At, and 11.9 m for Do	(109, 110)
FIR study	NSCLC pts with treated and asymptomatic BM	P	Atezolizumab (At)	≥ 2	None	13	23	NR	2.5 m	6.8 m	(111)
JAVELIN Lung 200	NSCLC pts with locally treated and asymptomatic BM	P	Avelumab (Av)	1–3	Docetaxel	46 Av, and 33 Do	In PD-L1+ pts 18.9 for Av, and 10.6 for Do	NR	NR	NR	(112, 113)
Goldberg SB, et al.	NSCLC with asymptomatic BM	P	Pembrolizumab	≥ 1	None	37	18.9	29.4	1.9 m	9.9 m	(114, 115)
Wakuda K, et al.	NSCLC with treated and untreated BM	R	Pembrolizumab	1	None	23	Pts with treated BM 54, pts with untreated BM 60	77 for pts with treated BM, and 60 for pts with untreated BM	6.5 m for patients with treated BM, and 5.3 m for patients with untreated BM	21.6 m for the whole BM population	(116)
Crino L, et al	Non-squamous NSCLC with asymptomatic BM	P	Nivolumab	> 1	None	409	19	NR	3 m	8.6 m	(117)
Dudnkik E, et al.	NSCLC	R	Nivolumab	1–2	None	5	40	40	NR	NR	(118)
Watanabe H, et al	NSCLC	R	Nivolumab	NR	None	19 with CNS mets, 29 without CNS mets	11 with CNS mets, 17 without CNS mets	NR	1.8 m for pts with CNS mets, and 3.63 m for pts without CNS mets	NR	(119)
Debieuvre D et al,	NSCLC with baseline BM	R	Nivolumab	> 1	None	477	NR	NR	NR	9.7 m for pts with BM, and 11.9 m for pts without BM	(120)
Assié JB, et al	NSCLC	R	Nivolumab	NR	NR	1,800	NR	NR	NR	9.9 m	(121)
Debieuvre D et al,	NSCLC with baseline BM	R	Nivolumab	> 1	None	477	NR	NR	NR	9.7 m for pts with BM, and 11.9 m for pts without BM	(120)
Grossi F, et al.	Non-squamous NSCLC	R	Nivoumab	> 1	None	409	17	NR	3 m	8.6 m	(122)
Cortinovis D, et al	Squamous NSCLC	R	Nivolumab	≥ 1	None	37	10	NR	4.9 m	5.8 m	(123)
Studies with ICI-ICI combinations in NSCLC											
CheckMate 227	NSCLC with treated asymptomatic BM	P	Nivolumab and ipilimumab (NI)	1	Chemotherapy (ChT)	69 NI and 66 ChT	33 NI and 26 ChT	NR	5.4 m for NI and 5.8 m for ChT	18.8 m for NI and 13.7 m for ChT	(124)
Hendriks LE, et al	NSCLC	R	Anti–PD-1/PD-L1 ± anti–CTLA-4	1–8	None	255	20.6% with BM vs. 27.7% without BM	27.3	1.7 m for pts with BM, and 2.1 m for pts without BM	8.6 m for pts with BM, and 11.4 m for pts without BM	(125)
Studies with ICI-chemotherapy combinations in NSCLC											
ATEZO-BRAIN	Non-squamous NSCLC with untreated BM	P	Atezolizumab, carboplatin and pemetrexed	1	None	40	47.5	40	8.9 m	13.6 m	(126)
KEYNOTE-189	Non-squamous NSCLC with asymptomatic BM	P	Pembrolizumab, platinum and pemetrexed (PPP)	1	Platinum and pemetrexed (ChT)	73 PPP, and 35 ChT	47.6 for PPP and 18.9 for ChT	NR	6.9 m for PPP, and 4.7 for ChT	19.2 m for PPP and 7.5 m for ChT	(127, 128)
Sun L, et al.	NSCLC with treated and untreated BM	R	Pembrolizumab ± chemotherapy	≥ 1	None	22	27.8	36.4	9.2 m	18 m	(129)
KEYNOTE-021, KEYNOTE-189, and KEYNOTE-407	NSCLC	R	Pembrolizumab, plus chemotherapy (PChT)	1	Platinum and pemetrexted (ChT)	171: PChT, 1127: ChT	For pts with BM: 39% for PChT, and 19.7% for ChT	NR	6.9 m for PChT, and 4.1 m for ChT	18.8 m for PChT, and 7.6 m for ChT	(130)
CheckMate 9LA	NSCLC pts with treated asymptomatic CNS BM	P	Nivolumab, ipilimumab and chemotherapy (NICH)	1	Chemotherapy (ChT)	64 NICH, and 58 ChT	38 for NICH, and 25.4 for ChT	NR	NR	19.9 m for NICH, and 7.9 m for ChT	(131, 132)
Studies with ICI-chemotherapy combinations in SCLC											
IMpower 133	SCLC with treated asymptomatic CNS metastases	P	Carboplatin, etoposide and atezolizumab (CEA)	1	Carboplatin and etoposide (CE)	17 CEA, and 18 CE	60.2 for CEA, and 65.4 for CE	NR	NR	NR	(133)
CASPIAN	SCLC with untreated asymptomatic or treated and stable BM	P	Platinum, etoposide and durvalumab (PED)	1	Platinum and etoposide (PE)	28 PED, and 27 PE	68 for PED and 58 for PE	NR	4.7 m for PED and 4.5 for PE	11.7 for PED, and 8.8 for PE	(134, 135)
Studies with cranial radiotherapy with and without immunotherapy in NSCLC											
Hubbeling H G, et al.	NSCLC with BM	R	SRT ± ICI (administered before or concurrently)	≥1	None	50: RT + ICI, 113 RT	NR	NR	NR	NR	(136)
Schapira E, et al.	NSCLC with BM	R	SRS + anti-PD-1/PD-L1 inhibitors, concurrent and sequential	NR	None	37	NR	NR	NR	17.6 m	(137)
Eright TL, et al.	NSCLC with BM	R	SRT ± anti-PD-1/PD-L1	NR	None	SRT 44, SRT + ICI 33	NR	NR	NR	13.9 m	(138)
Ahmed KA, et al.	NSCLC with BM	R	RT (SRS or FSRT) ± anti–PD-1/PD-L1	≥1	None	17	NR	NR	NR	17.9 m	(139)
Soccianti S, et al.	NSCLC with BM	R	RT (SRS, SRT or HFSRT) with or without ICI concurrent and sequential	NR	None	RT + ICI 100, RT 50	NR	NR	NR	1-year OS: SRT + ICI64.5%, and for SRT: 67.5%	(140)

ORR, objective response ratio; ICR, intracranial response ratio; DCR, disease control rate; PFS, progression-free survival; OS, overall survival; mOS, median overall survival; NR, not reported; m, months; pts, patients; mets, metastases; RT, radiotherapy; WBRT, whole brain radiotherapy; PBI, partial brain irradiation; SRS, stereotactic radiosurgery; SRT, stereotactic radiotherapy; FSRT, fractioned stereotactic radiation therapy; HFSRT, hypofractioned SRT.

**Figure 3:**
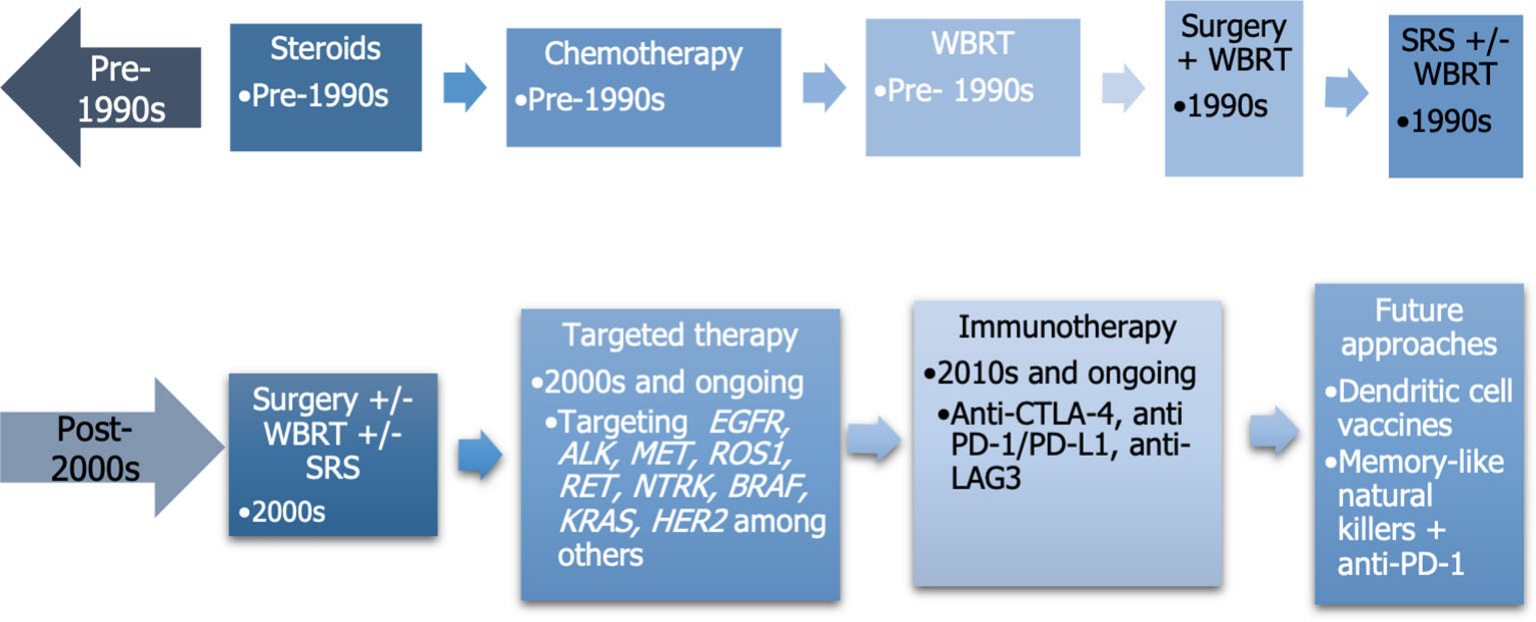
Temporal evolution of advancements used to treat brain metastases. WBRT, whole brain radiotherapy; SRS, stereotactic radiosurgery.

The development of different therapies for lung cancer and BM can be represented in a timeline, as depicted in **Figure 3** (142, 143).

### Studies with immune checkpoint monotherapy in NSCLC

#### *Atezolizumab: clinical trials

Among the different clinical trials evaluating ICI monotherapy, the OAK study included 1,225 pretreated NSCLC patients and allowed for the inclusion of patients with treated and asymptomatic CNS BM (*n* = 123). The study compared atezolizumab versus docetaxel. Patients with BM who were treated with atezolizumab exhibited a trend toward improved OS compared to those who received docetaxel. The OS were 16 and 11.9 months (HR = 0.74, 95% CI: 0.49–1.13; *p* = 0.1633), for the atezolizumab and docetaxel groups, respectively, in patients with BM. OS estimates at 2 years were 26.6% (95% CI: 15.1–38.1) in the atezolizumab arm and 19.3% (95% CI: 8.2–30.4) in the docetaxel arm in patients with BM (109). The FIR study was a single-arm trial, which evaluated the effect of atezolizumab in 138 previously treated NSCLC patients, with PD-L1–selected tumors (i.e., >5% PD-L1 staining in TC or IC) and allowed the inclusion of patients with treated and asymptomatic BM (*n* = 13). Patients with BM had an objective response rate (ORR) of 23% (range: 5–54), the median progression-free survival (mPFS) was 2.5 months (range: 1.0–11.3), and the OS was 6.8 months (range: 3.2–19.4). The study found that the activity of atezolizumab regarding ORR and OS was similar between patients with and without BM (111).

#### *Pembrolizumab: clinical trial

A study by Goldberg et al, evaluated the use of pembrolizumab in pretreated patients with NSCLC and untreated BM. In 37 patients with a PD-L1 expression of at least 1% in mononuclear inflammatory cells and tumor cells, the ORR was 18.9% (95% CI: 8.0–35.1), the mPFS was 9 months (95% CI 1.8–3.7), the median OS (mOS) was 9.9 months (95% CI: 7.5–29.8), and the estimated 2-year OS was 34% (range: 21–54) (114, 144).

#### *Pembrolizumab: retrospective study

Wakuda et al. studied the effect of pembrolizumab in treatment naïve NSCLC patients with PD-L1 TPS ≥50, with treated and untreated BM (*n* = 23) and without BM (*n* = 64). The study found no significant differences regarding ORR in patients with and without BM (54% and 60%, *p* = 0.77). However, patients with treated BM had an ORR of 77%, whereas patients with untreated BM had an ORR of 60% (*p* = 0.21) mPFS were 6.5 months (95% CI: 0.5–not reached), and 5.3 months (95% CI: 0.4–10.8) for patients with and without BM (116).

#### *Nivolumab: prospective studies

An Italian study derived from an expanded access program evaluated the use of nivolumab in 1,588 pretreated patients with non-squamous NSCLC, with unselected PD-L1, and allowed the inclusion of patients with BM if they were asymptomatic (*n* = 409). The ORR for patients with BM was 17%, the disease control rate (DCR) was 39%, the mPFS was 3 months (95% CI: 2.7–3.3), and the mOS was 8.6 months (95% CI: 6.4–10.8) (117).

#### *Nivolumab: retrospective studies

A study by Watanabe et al. evaluated nivolumab in 48 patients with NSCLC with and without CNS metastases. The study found a systemic ORR for patients with CNS metastases of 11% and 17% for patients without BM; mPFS were 1.8 months and 3.63 months, respectively. Another study by Debieuvre et al, including 2,858 patients with pretreated NSCLC, evaluated the effect of nivolumab; the study included 477 patients with baseline BM. The study did not find differences regarding OS in patients with or without BM (*p* > 0.05) (120). A different study by Assié et al. evaluated the effect of nivolumab in 10,452 NSCLC patients, including 1,800 with baseline BM. The study found that patients with BMs tended to be younger, more frequently female, with a non-squamous tumor histology and with higher prevalence of malnourishment compared to those without BM. The mOS for patients with BM was 9.9 months (range: 9–10.9), compared to 11.7 months (range: 11.3–12.2) in the overall population (121). An Italian study by Grossi et al, in 1,588 previously treated PD-L1 unselected non-squamous NSCLC patients, including 409 patients with asymptomatic and controlled CNS metastases, evaluated the efficacy of nivolumab. Patients with BM had an ORR and DCR of 17% and 39%, whereas the mPFS and mOS were 3 months (95% CI: 2.7–3.3) and 8.6 months (95% CI: 6.4–10.8), respectively. The study found that patients with BM had higher rates of liver metastases and had received two or more prior lines of therapy, both of which were statistically significantly different from patients without BM (117). A different Italian study by Cortinovis et al. studied the effect of nivolumab in 37 previously treated squamous NSCLC patients with CNS metastases. The study found an ORR of 19%, an mPFS of 4.9 months (95% CI: 2.7–7.1), and a mOS of 5.8 months (95% CI: 1.9–9.8).

As previously described, treatment with ICI monotherapy is an effective therapy for NSCLC patients with BM, however the only prospective evidence comes from the trial by Goldberg et al, in which pembrolizumab demonstrated a benefit in this subset of patients. Regarding both retrospective and prospective studies, PFS and OS ranged from 1.9 to 4.9 months and 5.8 to 21.6 months for patients with BM, respectively. It is worth noting that patients with BM treated locally, who were systemic treatment naïve at the start of the ICI therapy were the ones who had obtained the best survival outcomes. However, these data must be analyzed cautiously, since each study had a different design and the baseline characteristics of the patients were different.

### Studies with immunotherapy-immunotherapy combinations in NSCLC

Other treatment strategies involve the use of immunotherapy–immunotherapy combinations; the CheckMate 227 clinical trial evaluated the effect of nivolumab plus ipilimumab compared to chemotherapy in 1,166 chemotherapy-naïve NSCLC patients. Inclusion was based on a TMB of at least 10 mt/Mb, regardless of PD-L1 expression, patients with treated asymptomatic BM were allowed (*n* = 135). The ORR was 33% and 26%, mPFS 5.4 months (3.1–8.6) and 5.8 months (4.3–8.0) with a HR (95% CI) of 0.79 (0.52–1.19), and mOS 18.8 months (9.2–29.4), and 13.7 months (10.5–16.2) with a HR (95% CI) of 0.57 (0.38–0.85), for nivolumab plus ipilimumab and chemotherapy, respectively (124). A study by Hendriks et al. evaluated the use of ICI monotherapy or ICI combinations in 1,025 patients with NSCLC, of which 255 had BM, including active and symptomatic. Patients with and without BM had an ORR of 20.6% and 22.7% (*p* = 0.484) and a DCR of 43.9% and 52% (*p* = 0.024), respectively; the intracranial response of patients with active BM was 27.3%. The mPFS for patients with and without BM were 1.7 months (95% CI: 1.5–2.1) and 2.1 months (95% CI: 1.9–2.5) (*p* = 0.009), whereas the mOS were 8.6 months (95% CI: 6.8–12), and 11.4 months (95% CI: 8.6–13.8) (*p* = 0.035), respectively. Patients who previously received cranial radiotherapy did not have a different outcome in terms of OS compared to those who did not (125).

Although a smaller number of patients was included in ICI–ICI combination studies, there seems to be a greater benefit than with ICI monotherapy; however a conclusion should not be drawn since the populations compared were not the same.

### Studies with ICI plus chemotherapy in NSCLC

#### *Atezolizumab plus chemotherapy

Different clinical trials have evaluated the effect of immunotherapy-plus chemotherapy in the treatment of NSCLC patients, including those with BM. The study ATEZO-BRAIN evaluated the effect of atezolizumab, carboplatin, and pemetrexed in 40 previously untreated non-squamous NSCLC patients with untreated BM and unselected PD-L1 expression. The study reported an intracranial response of 40%, a systemic response in 47.5% of the patients; the median systemic PFS was 8.9 months (6.7–13.8), intracranial PFS was 6.9 months (4.7–11.9), and mOS was 13.6 months (9.72–not reached). Patients with a positive PD-L1 tended to present an increased benefit regarding survival (126).

#### *Pembrolizumab plus chemotherapy: prospective studies

The KEYNOTE-189 clinical trial evaluated the effect of the combination of pembrolizumab, platinum-based chemotherapy, and pemetrexed compared to placebo, platinum-based chemotherapy, and pemetrexed in 616 previously untreated non-squamous NSCLC patients. The study allowed for any level of PD-L1 expression and the inclusion of patients with asymptomatic BM (*n* = 108). Patients with BM had mPFS of 6.9 months (5.4–11.0) and 4.7 months(2.2-5.5), HR: 0.42 7.5 and the mOS were 19.2 months (15.0–25.9) and 7.5 months (4.6–10.0), HR: 0.41 (0.24–0.67) for the pembrolizumab and the placebo combinations, respectively (127).

#### *Pembrolizumab plus chemotherapy: retrospective studies

A study by Sun et al. evaluated the effects of pembrolizumab alone or in combination with chemotherapy in patients with NSCLC with (*n* = 126) and without BM (*n* = 444); the study included patients with treated and untreated BM. Patients with BM had an intracranial ORR of 36.4%; the systemic ORR was 27.8% for patients with BM and 29.7% for patients without BM (*p* = 0.671). The estimated mPFS for patients with BM was 9.2 months, and for patients without BM was 7.7 months (*p* = 0.609); the estimated mOS were 18 and 18.7 months (*p* = 0.966), respectively. The study found that patients with treated BM had a significantly improved mPFS and mOS compared to those with untreated BM. However, the study did not report the differences in terms of response or survival comparing pembrolizumab monotherapy versus pembrolizumab plus chemotherapy (129). A pooled analysis of the KEYNOTE 021, 189, and 407 clinical trials by Powell et al. evaluated the effect of platinum-based chemotherapy with or without pembrolizumab, in 1,298 treatment naïve patients with NSCLC and baseline BM (*n* = 171) including treated and stable or untreated and asymptomatic BM. Patients with BM who received pembrolizumab based treatment had an ORR of 39% (95% CI: 29.7–49.1), compared to 19.7% (95% CI: 10.9–31.3%) in patients who received the chemotherapy-based treatment, the mPFS were 6.9 months (95% CI: 5.7–8.9) and 4.1 months (95% CI: 2.3–4.6) for each group, respectively, with a HR: 0.44 (95% CI: 0.31–0.62). The mOS were 18.8 months (95% CI: 13.8–25.9) and 7.6 months (95% CI: 5.4–10.9) with a HR for death of 0.48 (95% CI: 0.32–0.70) for the pembrolizumab-plus chemotherapy and chemotherapy treatment groups. Patients without BM had a better ORR, mPFS, and mOS than patients with BM in both treatment groups.

#### *Nivolumab and ipilimumab plus chemotherapy: prospective studies

A different strategy was evaluated in the CheckMate 9LA, in which 1,150 treatment naïve NSCLC patients, were randomized to receive nivolumab, plus ipilimumab with two cycles of chemotherapy *versus* four cycles of chemotherapy alone. The study allowed for any expression of PD-L1 and for patients with treated asymptomatic CNS metastases. The ORRs for the whole population were 38.2% (33.2–43.5) and 24.9% (20.5–29.7); the mOS for patients with CNS metastases was 19.9 months versus 7.9 months, HR: 0.47 (95% CI: 0.31–0.71), for the immunotherapy-based treatment and the chemotherapy arm, respectively (131).

Both prospective and retrospective studies show a clear benefit in the use of chemoimmunotherapy combinations for patients with NSCLC and BM, regardless of the ICI used.

Results from these trials suggest that this combination therapy might be more effective than ICI monotherapy, however, as previously stated comparisons among different trials should not be performed.

### Chemoimmunotherapy in patients with SCLC

Studies evaluating the effect of chemoimmunotherapy in SCLC patients allowing the inclusion of BM include the IMpower 133 and the CASPIAN clinical trial.

#### *Atezolizumab plus chemotherapy: prospective study

The IMpower133 evaluated the use of atezolizumab, carboplatin, and etoposide, compared to carboplatin and etoposide in 403 treatment naïve SCLC patients, allowing the inclusion of patients with treated asymptomatic BM (*n* = 35). The study reported that patients with BM had no different outcomes regarding PFS and OS regardless of the treatment, with a HR for death (95% CI) of 1.07 (0.47–2.43) (145).

#### *Durvalumab plus chemotherapy: prospective study

The CASPIAN clinical trial evaluated the use of durvalumab in combination with platinum-based chemotherapy versus platinum-based chemotherapy in 268 treatment naïve SCLC. Patients with untreated asymptomatic or treated and stable BM were allowed (*n* = 55), only patients in the chemotherapy-based arm were allowed to receive PCI. The mPFS was 4.7 months (range: 4.4–6.4) for the immunotherapy-based arm and 4.5 months (range: 4.5–5.9) for the chemotherapy-based arm, with an unstratified HR of 0.73 (95% CI: 0.42–1.29). The mOS for the durvalumab group was 11.7 months (8.3–16.3), and 8.8 months (5.7–11.9) for the chemotherapy arm, with an unstratified HR of 0.79 (95% CI: 0.44–1.41). The study reported that the durvalumab arm prolonged OS in patients with and without BM (134, 135). A meta-analysis studied the addition of ICI to chemotherapy versus chemotherapy as monotherapy in the first treatment of extensive stage SCLC; the study found that the only OS benefit from the addition of ICI was in patients without BM at diagnosis. These results are possibly associated to the fact that there is few information available for patients with BM (146). Larger real world data studies are expected to report the efficacy of chemoimmunotherapy combinations in SCLC patients with BM.

### Retrospective studies using cranial radiotherapy with and without immunotherapy in NSCLC

Combination strategies with radiotherapy and immunotherapy could lead to a synergy through the release of danger associated molecular patterns (DAMPs) and cytokines, thus increasing neoantigen presentation and diversifying the T-cell repertoire. However, combining these therapies could also potentiate the incidence of adverse events. Different retrospective studies have evaluated the effect of these therapy combinations in BM from NSCLC (147). A study by Hubbeling et al. included 163 patients with NSCLC and BM who received either radiotherapy alone (*n* = 113) or in combination with ICI (*n* = 50). In patients who received both treatments, radiotherapy was more frequently administered prior to ICI, compared to concurrent administration. The study reported similar rates of radiotherapy related toxicity in both groups, thus, suggesting that ICI administered with radiotherapy may not be related to an increased the risk of toxicity (136). In another study by Schapira et al, 37 patients with NSCLC patients and BM were evaluated to assess the effect of the treatment with anti–PD-1 inhibitors and stereotactic radiosurgery (SRS). The study found that patients treated concurrently with ICI and SRS had an improved OS, compared to those treated with SRS before or after ICI, the 1-year OS were 87.3%, 70.0%, and 0% (*p* = 0.008) for each treatment, respectively. Distant brain failure (i.e., the emergence of a new BM or tumor progression outside the prior radiation treatment field in the brain) was lower in patients treated with concurrent SRS and ICI, compared to those with sequential treatment. The study found a numerically higher frequency of radiation associated toxicities in patients who received the concurrent treatment (137, 148). A different study by Enright et al. evaluated the use of SRT combined with (*n* = 33), or without (*n* = 44) ICI in NSCLC patients with BM. Patients who received both treatments had a decreased distant brain failure, decreased rates of death by neurological causes, as well as a better OS compared to patients who received radiotherapy without ICI; the mOS for the global cohort was 13.9 months (range: 1.1–61.6). Regarding toxicities, symptomatic brain radionecrosis was more frequent in patients receiving radiotherapy alone (11.3%) compared to ICI plus radiotherapy (6%) (138). Another study by Ahmed et al. evaluated the use of SRT with or without anti–PD-1/PD-L1 inhibitors in 17 patients with NSCLC and BM; the study included patients who received SRT prior, concurrent, or after ICI. Administration of radiotherapy concurrent or posterior to ICI was associated with an improved OS (*p* = 0.006) compared to prior ICI; the mOS for the entire cohort was 17.9 months (139). The study ARIO evaluated the effect of radiotherapy with (*n* = 100) or without ICI (*n* = 50) in patients with NSCLC and BM. The study found that patients receiving the treatment combination had a longer intracranial local PFS (*p* = 0.007); however, no differences were observed in terms of OS. The study found that patients with non-adenocarcinoma histology tumors and a KPS of 70 were associated with a worse OS. Furthermore, patients who received ICI and radiotherapy within ≤7 days of each treatment had an improved OS. The study did not find differences regarding rates of radionecrosis between treatments (140). The evidence derived from these studies highlight the beneficial synergic activity of ICI plus radiotherapy, rendering it a safe and tolerable procedure. Furthermore, it seems that timing in this context is important, since patients receiving radiotherapy (SRS/SRT) either concurrent or posterior to the start of ICI derive the greatest benefit. Additionally, most of the studies reflect that concurrent administration of these treatments leads to an improved OS.

## Resistance to treatment strategies

### General mechanisms of resistance to immunotherapy

Different mechanisms of resistance to immunotherapy have been identified, not exclusively in the context of BM. These include cancer cell–related mechanisms, such as the loss of neoantigen production, which could be triggered by hypermethylation in the promoter region of genes coding for neoantigens. Another described mechanism is alterations in neoantigen presentation, due to alterations in HLA class 1 genes, loss of function of β2-microglobulin, derangements in proteins related to antigen degradation, as well as disruptions in the IFN-JAK-STAT signaling pathway. Alterations in the latter can be associated with mutations and copy-number variations in the IFN pathway. Other identified mechanisms include those associated with a loss of function of JAK1 and JAK2 (149). Immune cell-associated mechanisms of resistance to ICI can be attributed to tumors that lack TILs, particularly CD8+ T cells. These tumors do not engage in antigen presentation and cannot trigger an inflammatory response. A number of mechanisms have been proposed to explain the absence of TILs in tumors, including tumor signaling through the β-catenin pathway, as well as loss of certain genes such as PTEN, SKT11 (LKB1), and KEAP1, among others. Overall, these alterations lead to an immunosuppressive environment, with impaired inflammatory signaling, which can lead to resistance to ICI. Another mechanism associated with resistance to ICI is the location of the tumor, such as liver metastases, which have traditionally been associated with an immunosuppressive environment. Other immune cells that are associated with an immunosuppressive TME include MDSCs and TAM-M2 cells, among others (149).

### Particular mechanisms of resistance to immunotherapy in BM

A proposed hypothesis is that there is tumor heterogeneity in BM, with different genomic and epigenomic alterations from the primary tumor (as reviewed previously), and among the BMs. In fact, tumors, both primary and metastases, are composed of different subclones with different targets, as well as different mechanisms of resistance (35). As previously stated, there exists a significant heterogeneity regarding the expression of PD-L1 and TMB according to tumor sample sites; thus, tumor heterogeneity of BM can be associated to resistance to immunotherapies.

It has been suggested that metabolic derangements in substrate consumption by tumor cells can lead to ineffective activity of the immune cells in the TME. These events are associated with a metabolic struggle of the tumor infiltrating immune cells. Both T cells and tumor cells heavily depend on glucose for their energy needs. Tumor cells, owing to their rapid growth and energy requirements, predominantly engage in aerobic glycolysis to process glucose. This leads to the generation of high levels of lactic acid in the TME, which reduces the availability of glucose for immune cells, resulting in compromised T cell function as well as a heightened recruitment of Tregs, and polarizes microglial cells and TAMs toward a pro-tumorigenic state. Furthermore, there is a competition for amino acids such as glutamine, glutamate, and tryptophan among T cells, MDSCs, and tumor cells. Within the TME, substances such as kynurenine, produced by the tumor cells, MDSCs, and TAMs, hinder the activation of T cells and promote the generation of immune-suppressive Tregs. The brain microenvironment provides ample glutamine and tryptophan, facilitating tumor cells in adapting and utilizing these amino acids for their growth. Moreover, the accumulation of lactic acid in the TME boosts the expression of PD-1 on T cells and PD-L1 on tumor cells, resulting in the suppression of immune cell function (150). These immune contextures of the brain might require specific therapeutic approaches when treating patients with NSCLC and BMs.

## Future perspectives

Different biomarkers have been described and are currently being developed to detect and characterize genomic, molecular, or TME alterations in BM, as biomarkers of prognosis or targeted treatment. As such, detection of circulating tumor DNA (ctDNA) in the cerebrospinal fluid (CSF) can be an attractive strategy and less invasive than conventional biopsies. ctDNA allows for the characterization of different genomic alterations within CNS metastases and provides a landscape of tumor heterogeneity (151). High TMB has been associated with response to ICIs, and high TMB has been detected in BM (89). Using TMB as a biomarker of response is an attractive strategy, since it can also be detected in ctDNA from CSF (152). However, there is no clinical evidence that a high TMB in BM correlates to a benefit from ICIs, and the use of ctDNA from CSF in patients with BM is not currently a standard practice.

The most validated biomarker associated with response to ICIs is detection of PD-L1, either in tumor cells, immune cells, or both and to a lesser extent TMB. High expression of both of these biomarkers has been associated with an increased survival in NSCLC patients treated with ICI (153). As previously presented TILs in BM have been associated with an improved survival in patients with NSCLC and SCLC (81, 83) and could potentially serve as biomarkers of benefit. In addition, a different biomarker is a high neutrophil-to-lymphocyte ratio, which has been associated with a worse prognosis in patients being treated with radiosurgery or neurosurgery for BM (154, 155).

Although not particularly associated with BM, an increased representation of different bacteria from the gut microbiota has been detected in the stool of patients who responded to treatment with ICI or ICI-based therapies. Among the most representative bacteria, *B longum*, *E hirae*, *A muchiniphila*, *B fragilis*, *Collinsella aerofaciens*, and *F prausnitzii* stand out as biomarkers associated with response (156–161). Furthermore, clinical trials in melanoma patients have performed fecal microbiota transplantation (FMT) from patients who received ICI and responded to patients with who received the same treatment and did not respond. Patients with primary resistance to ICI who received a FMT from responders had an approximate 20% rate of response following the FMT and continuing treatment with ICI. These results show that manipulation of the gut microbiome through FMT is a strategy useful to overcome resistance to ICI (162, 163).

Regarding future therapeutic strategies, targeting other regulatory checkpoints, such as LAG3, TIGIT, or TIM3, either alone or in combination with anti–PD-1/PD-L1 inhibitors are being explored as means to overcome resistance to current immunotherapies. Many clinical trials with different combinations are currently undergoing. Furthermore, suppression of immune cells of molecules that elicit an immunosuppressive environment could also be a target, such as M2-TAMs, MDSC, and anti-TGFβ treatments. Other strategies are being used in NSCLC patients with BM, such as clinical trials involving vaccines with mRNA tumor antigens pulsed into dendritic cells (NCT02808416) or administration of autologous dendritic cells to the tumor through the intrathecal space (NCT03638765). Results from these studies are expected to provide a new strategy to treat these patients.

## Concluding remarks

BMs represent a devastating event in patients with lung cancer, with alarming numbers regarding its incidence. The development and establishment of BMs is a complex process that needs to be studied from different perspectives, including genetic, epigenetic, immunologic, physiologic, and those associated with the BBB and the brain parenchyma. The cascade of signaling events leading to BMs is complex, and still not completely understood, in part due to a limited access to BMs samples, and the heterogeneity among these tumors. Immunotherapy represents a milestone in the treatment of patients with lung cancer, and it has clearly demonstrated that either alone or in combination with chemotherapy or radiotherapy, patients with BM derive a benefit. However, selecting patients who will benefit from one combination of therapies or another, continues to represent a challenge, and so does the sequence of treatment administration. Although important breakthroughs have been achieved with the arrival of immunotherapy, patients with lung cancer and BMs still present a deleterious diagnosis which needs to be approached by a multidisciplinary team and requires further research.

## Author contributions

AR-H performed the literature search and developed the outline as well as the initial draft. EA edited the manuscript, added further content and provided supervision. All authors contributed to the article and approved the submitted version.
